# Low and High Doses of Espresso Coffee Improve Repeated Sprint Performance and Eye–Hand Coordination Following Fatigue Status in Male Basketball Players

**DOI:** 10.1016/j.cdnut.2024.104427

**Published:** 2024-07-25

**Authors:** Alireza Niknam, Mohammad Hasan Abdullahi, Mohammad Hemmatinafar, Amir Hossein Alaeifar, Maryam Koushkie Jahromi

**Affiliations:** 1 Department of Sports Sciences, School of Education and Psychology, Shiraz University, Shiraz, Iran; 2 Department of Sports Sciences, Faculty of Physical Education, Karaj Payam Noor University, Karaj, Iran

**Keywords:** coffee, repeated sprint, coordination, fatigue, basketball

## Abstract

**Background:**

Although several studies have evaluated the effect of coffee on sports performance, the effect of caffeine on sports performance during fatigue status remains unclear.

**Objectives:**

This study aimed to determine the effect of high and low doses of coffee on the repeated sprint test (RST), perceived fatigue (PF), and eye–hand coordination following physical fatigue status in male basketball players.

**Methods:**

Twenty-four male basketball players were randomly placed in 4 conditions including *1*) low-dose espresso coffee (LDEC); *2*) high-dose espresso coffee (HDEC); *3*) decaffeinated espresso coffee (PLA); and *4*) no drinking (CON). PF and eye–hand coordination were measured using the soda pop test (SPT) at baseline, immediately after the RST, and 5 min after the 10 all-out sprints with a 30-s interval of RST.

**Results:**

The time of the first to tenth sprints (RST_1_ to RST_10_), total time (RST-TT), mean time (RST-MT), best time (RST-BT), and percentage of performance decrement (PD) were recorded. Coffee dose-dependently significantly improved RST-TT, RST-MT, and RST-BT compared with PLA and CON. PF increased significantly in all conditions immediately after RST compared with baseline. Five minutes after RST, PF was reduced compared to immediately after RST. Immediately after RST, coffee reduced PF dose-dependently compared with PLA and CON. SPT decreased immediately after RST in PLA and CON compared with baseline, whereas no significant change was observed for LDEC and HDEC. At baseline and immediately after RST, coffee and placebo consumption increased SPT performance compared with CON. Immediately and 5 min after RST, coffee increased SPT performance compared to PLA dose-dependently.

**Conclusions:**

HDEC and LDEC improved RST performance and eye–hand coordination in male basketball players. However, HDEC showed a more profound effect compared with LDEC.

## Introduction

Optimal performance in basketball requires a high level of biomotor and skill abilities [[1], [2], [3], [4]]. During a basketball game, players perform ∼105 high-intensity (2–6 s) efforts (including sprints, jumps, throws, etc.) every 21 s [5]. However, the quantity and quality of high-intensity efforts appear to decrease in the last minutes of each game, which could be due to the development of fatigue [4,6]. Hence, reduced ability to perform high-intensity efforts during competition or training limits not only individual physical capacities but also team performance [7]. Therefore, basketball players should be able to perform skills such as eye–hand coordination, jumping, and throwing optimally while maintaining the quality of performance in repeated sprints.

It has been suggested that nutritional strategies such as acute consumption of some ergogenic aids (approved by the World Anti-Doping Agency) may reduce fatigue or its detrimental effect on performance. Therefore, the consumption of supplements or ergogenic drinks is common in athletes to reduce fatigue, increase performance, and optimize recovery [8]. One of the most common ergogenic aids is caffeine and caffeinated coffee [9,10]. Caffeine is a stimulant alkaloid that is rapidly metabolized in the liver and converted into 3 types of dimethylxanthine (paraxanthine, theophylline, and theobromine), which remain in the blood for a longer time than caffeine [9]. Caffeine is soluble in water and fat and crosses most of the barriers in the body (blood-brain barrier, cerebrospinal fluid, and muscle cell membrane), so it is easily distributed to all organs or systems of the body [11,12]. Therefore, caffeine can have a significant effect on various body systems, including the neuromuscular, cardiovascular, and endocrine systems, which ultimately affects metabolism and sports performance [13].

Caffeine has been found to reduce symptoms of physical and mental fatigue and improve sports performance [13,14]. The effects of caffeine on sprint performance and neuromuscular coordination have been less investigated, and most studies have emphasized aerobic endurance and related mechanisms [10,13]. Additionally, most studies have examined anhydrous caffeine or sometimes energy drinks, chocolates, or jellies as a source of caffeine [10]. However, the benefits of coffee as the most widely used method of caffeine consumption worldwide [15] for improving the performance of athletes remain almost unknown [10]. Coffee, in addition to caffeine (the most well-known biological component of coffee), is a complex combination of bioactive substances such as vitamin B-3, magnesium, potassium, phytochemicals (caffeic acid and chlorogenic acid), lactones, diterpenes, niacin, and trigonelline [[16], [17], [18]], which may affect the biological function of caffeine or exercise performance. One study reported that anhydrous caffeine and coffee, standardized to provide 5 mg/kg of caffeine, were similarly effective in improving endurance performance [19]. Similar results have been reported for resistance and sprint exercises [20,21]. A study suggested that coffee can be a good source of caffeine before high-intensity interval training. Another study found that in trained male runners, 60 min after consuming 0.09 g/kg caffeinated coffee, 1-mile race performance increased by 1.9% and 1.3% compared with placebo and decaffeinated coffee, respectively [22]. Also, it has been reported that acute coffee consumption has significant functional benefits on reaction time in habitual caffeine users [23]. The deteriorating effect of fatigue on neuromuscular functions has been approved [24], whereas the role of caffeine on sports performance in fatigue status is not clear. Also, no study was found regarding the effect of different doses of coffee, especially on repeated sprint performance and eye–hand coordination of athletes, especially in basketball players. Therefore, the present study investigated the effect of different doses of espresso coffee on repeated sprint ability and eye–hand coordination performance during a fatigued state.

## Methods

### Participants

Twenty-four trained young male basketball players with ∼2 y of participation in a basketball league (semi-professional basketball players; age: 20.6± 1.7 y; height: 184.1 ± 4.9 cm; weight: 72.8 ± 3.3 kg; BMI: 21.5 ± 1.1 kg/m^2^) who were mild habitual coffee consumers volunteered to participate in this research. Caffeine consumption was assessed using a caffeine consumption questionnaire [25] and, based on the proposals by Filip et al. [26], was defined as <3 mg/kg/d mild habitual coffee consumption. The inclusion criteria were participation in official basketball competitions in the past 2 y, physical health with no injuries, no history of allergy to caffeine or coffee, and no history of smoking. Exclusion criteria included being affected by an infectious disease during the study (cold, flu, etc.), musculoskeletal injuries, and taking drugs or ergogenic supplements. Before initiating the intervention, the study procedures were explained to the participants, and they signed written informed consent. This study was reviewed and approved by the Ethics Committee of the Shiraz University, Shiraz, Iran (ID number: SPE/.IR.US.PSYEDU.REC.1402.002) and carried out according to the Declaration of Helsinki.

The number of participants in this study was determined based on the study by Goods et al. [27], according to which caffeine ingestion (3 mg/kg body weight) led to a significant improvement in sprint performance compared with placebo (Cohen’s *d* = 1). Using G∗Power 3.1, considering a confidence interval of 95% and analysis power of 0.90, it was determined that ≥11 participants were needed for this study. Karayigit et al. [28] also noted that coffee ingestion improved average peak power output in repeated sprint cycling (12 sprints) compared with placebo (pEta^2^ = 0.29). Considering a confidence interval of 95% and analysis power of 0.90, it was determined that ≥10 participants were needed for the present study. To ensure a sufficient sample size, 24 participants were selected for this study.

### Study design

**FIGURE 1:**
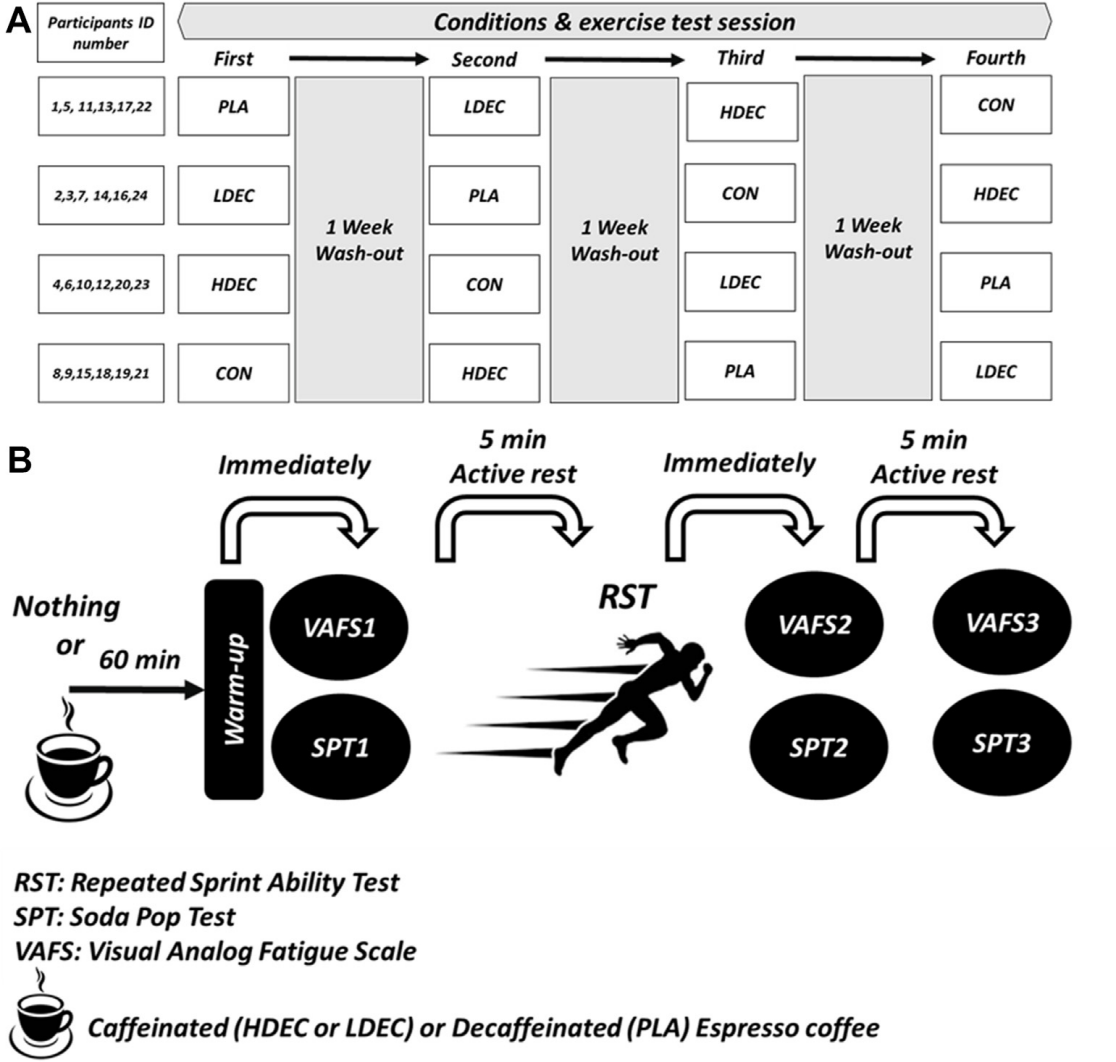
Study design. (A) Allocation of participants for each condition and exercise test session. (B) Arrangement and timing of tests for each session. CON, control (no coffee or placebo); HDEC, high-dose espresso coffee; LDEC, low-dose espresso coffee; PLA, placebo (decaffeinated espresso coffee).

This study was carried out in a randomized, crossover, placebo-controlled, and double-blinded design (Figure 1). One week before the beginning of the study, the participants underwent a familiarization session (to limit the effects of learning on the outcome of the experiment). During this session, participants were familiarized with all study procedures including testing protocols and performed the tests for familiarization. Then, participants performed 4 separate test sessions using the crossover method. In each test session, the participants were randomly placed in one of the conditions including *1*) decaffeinated espresso consumption as a placebo (PLA), *2*) low-dose espresso coffee (LDEC) consumption, *3*) high-dose espresso coffee (HDEC) consumption, and *4*) control without drinking anything (CON). According to previous studies, a 1-wk interval was considered as a washout period for each condition [[29], [30], [31]]. In each test session perceived fatigue was measured by visual analog scale (VAFS) [32,33] at baseline (VAFS1), immediately after the repeated sprint test (VAFS2), and 5 min after the repeated sprint test (VAFS). Soda pop test performance (SPT) was also measured at baseline (SPT1), immediately after the repeated sprint test (SPT2), and 5 minutes after the repeated sprint test (SPT3) (Figure 1). Sixty minutes before the exercise test session, participants drank coffee or a placebo under the supervision of trainers. Participants had to consume coffee/placebo from cups orally within a 5-min period. After an hour of passive rest, the warm-up was performed with the same protocol for each trial (5 min jogging, a series of sprint drills [10–15 s at each station: walking lunges, high knees, heel-flicks], and a series of dynamic stretches [3–4 min]). Immediately after warm-up, perceived fatigue and SPT performance were measured using the SPT (baseline). After 5 min of active rest (walking), each participant performed a repeated sprint test (RST) including 10 sprints × 30 m. Immediately and 5 min after RST, perceived fatigue and SPT performance were again measured.

All trials were completed at approximately the same time of day (between 10:00–11:00). All participants consumed the same breakfast containing 350 to 400 kcal (65% carbohydrates, 20% protein, and 15% fat) 2 h before the exercise test session. Participants were instructed to maintain their normal diet 12 h before the testing sessions, to avoid eating and drinking anything (except for water) 1 h before testing, and to avoid strenuous exercise 24 h before each trial. Participants were provided with a list of dietary sources of caffeine and asked to refrain from consuming them 12 h before each exercise test session, and this was verbally approved. In addition, participants had access to drink water on a self-selected basis during the trials.

### Preparation of drinks

The HDEC drink consisted of 18 g of caffeinated ground coffee (Lavazza Espresso Italiano, double shot). PLA was obtained from 18 g of decaffeinated ground coffee (Lavazza Espresso Decaffeinato, double shot). The LDEC drink was obtained from the combination of 9 g of caffeinated ground coffee and 9 g of decaffeinated ground coffee. The approximate amount of caffeine in LDEC, HDEC, and PLA was 80 mg, 160 mg, and 10 mg, respectively. The amount (2 oz) and temperature (82–88 °C) of the water, the pressure (9–10 bars: 900–1000 kPa) applied to make the coffee, and the espresso machine (Gevi 15 Bar 5403) were the same for preparing all drinks. HDEC, LDEC, and PLA groups consumed coffee drinks 1 h before beginning every exercise test session. Coffee was prepared by an independent third party blind to its consumption. The temperature of the drinks during consumption was so that it did not burn the mouth (∼71 °C).

### Repeated sprint test

The RST consisted of ten 30-m sprints performed every 30 s (on the basketball court). Two cones and marker lines were placed 30 m apart to indicate the sprint distance. The participants covered the distance between the 2 cones as fast as possible. Each sprint was initiated from a line 30 cm behind the start line (to prevent false triggering of the first timing gate). Computer-generated audio signals provided a 5-s countdown to the start of each sprint and subjects were verbally encouraged to give maximal effort. The time of each sprint (RST-ST_1-10_) was recorded with millisecond accuracy by stopwatch and speed gates with photoelectric sensors. Total (RST-TT), mean (RST-MT), and best (RST-BT) time during RST was recorded. Also, fatigue was calculated and recorded based on the percentage decrement (RST-PD) (Equation *1*) [34].

(1) Fatigue(%)=(100×(totalsprinttime÷idealsprinttime))−100

where total sprint time = sum of sprint times from all sprints and ideal time = number of sprints × fastest sprint time.

### SPT

The SPT was used to measure manual dexterity and eye–hand coordination. Previous studies have also used this test to determine eye–hand coordination [[35], [36], [37], [38]].

**FIGURE 2:**
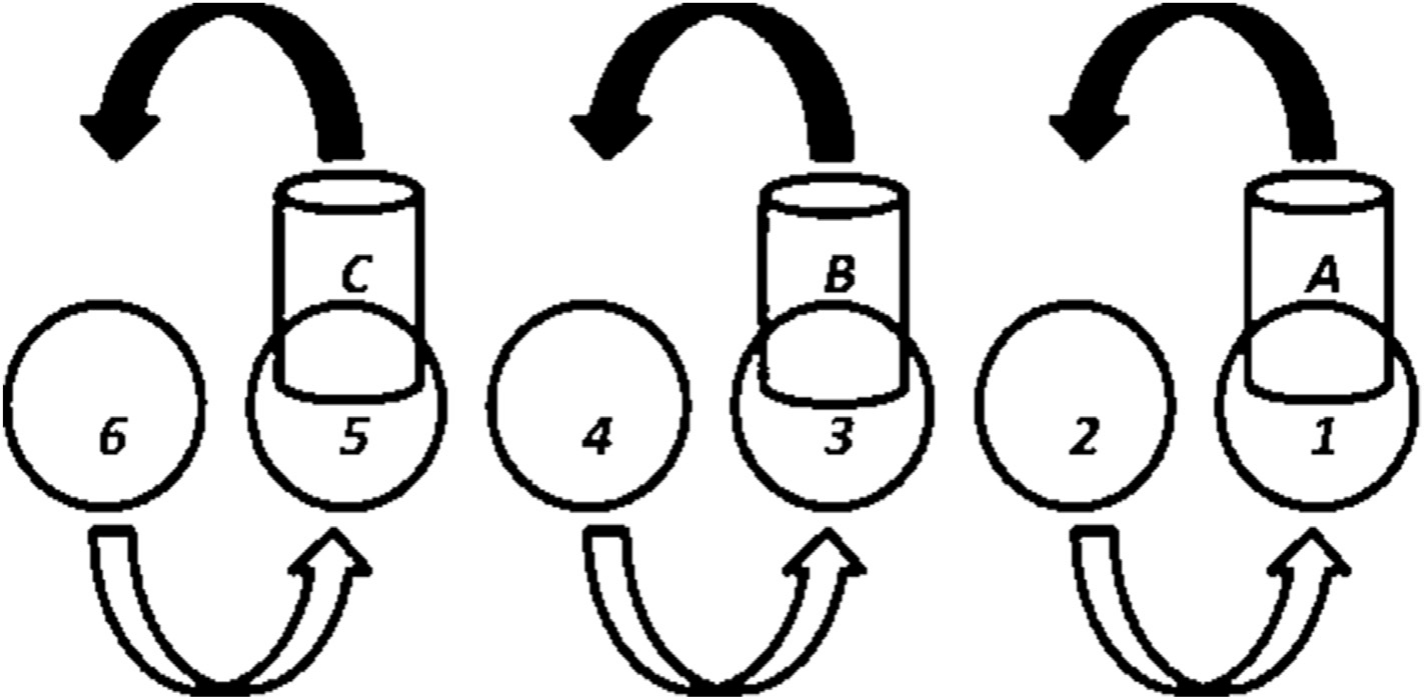
Soda pop test diagram.

Six circles (diameter: 8.26) with the same distance (3.81 cm) were drawn in a straight line centered on a board platform (dimensions: 81.28 cm in height and 12.7 cm wide). The full soda pop cans (355 mL) were placed in every other circle starting from the side of the hand being tested (dominant hand). Before initiating the test, the participants maintained a ready position by sitting behind the platform, with the hand in the “thumbs up” position and the elbow joint with ∼100° to 120° of flexion. To adopt a suitable ready position, a height-adjustable chair was used to sit behind the platform. With the command “go,” the test began, and the participants turned each soda can upside down and placed it in the empty circle on the side. Specifically, Can A was moved from circle 1 to circle 2, Can B was moved from circle 3 to circle 4, and Can C was moved from circle 5 to circle 6 (Figure 2). Then the participants returned the cans to their original positions in reverse order [37,39]. This process was repeated twice (as fast as possible), and the time was recorded with a stopwatch (Q&Q, HS43). It should be noted that the SPT test has an acceptable validity and reliability coefficient (0.81) [38,40].

### Statistics

All statistical analyses were conducted using the Statistical Package for the Social Sciences (SPSS for Windows, version 26). Measures of central tendency and spread were presented based on mean and SD. After confirming all assumptions, 1-way or multifactorial analysis of variance (ANOVA) or 1- and 2-way repeated measures ANOVA were used to determine the main effect for parametric data. Paired comparisons were performed using the Bonferroni post hoc test. α was set at 0.05 for all analyses.

## Results

### Perceived fatigue

Two-way (time × condition) repeated measures ANOVA showed that the main effect of time (*P* < 0.001), condition (*P* < 0.001), and interaction effect (*P* < 0.001) on perceived fatigue (VAFS) was significant (Table 1).

**TABLE 1:** Results of mixed repeated measures ANOVA test to determine the main effect of interventions on perceived fatigue, eye–hand coordination, and time of each sprint in repeated sprint test

Variables	Time/stage	CON (*n* = 24)	PLA (*n* = 24)	LDEC (*n* = 24)	HDEC (n = 24)	Main effects		
		Mean ± SD	Mean ± SD	Mean ± SD	Mean ± SD	Time	Condition	Interaction
Perceived fatigue (mm)	Baseline	4.5 ± 2.39	5.79 ± 3.69	5.58 ± 2.20	5.79 ± 3.24	F = 810.3 *P* < 0.001 η^2^ = 1	F = 27.1 *P* < 0.001 η^2^ = 0.5	F = 13.9 *P* < 0.001 η^2^ = 0.4
	Immediately after RST	68.04 ± 12.79	67.16 ± 11.12	58.95 ± 8.37	55.66 ± 8.68			
	5 min after RST	17.83 ± 7.79	19.5 ± 8.24	14.95 ± 7.45	15.91 ± 7.44			
Soda pop test (s)	Baseline	8.08 ± 0.44	8.00 ± 0.46	7.94 ± 0.47	7.88 ± 0.45	F = 22.8 *P* < 0.001 η^2^ = 0.5	F = 63.3 *P* < 0.001 η^2^ = 0.7	F = 32.6 *P* < 0.001 η^2^ = 0.6
	Immediately after RST	8.33 ± 0.5	8.12 ± 0.48	7.96 ± 0.47	7.87 ± 0.50			
	5 min after RST	8.02 ± 0.44	8.02 ± 0.43	7.90 ± 0.47	7.83 ± 0.48			
Time of each sprint (ST1-ST10) during the repeated sprint tests (s**)**	RST-ST1 (s)	5.56 ± 0.25	5.51 ± 0.29	5.49 ± 0.23	5.31 ± 0.18	F = 31.6 *P* < 0.001 η^2^ = 0.6	F = 21.7 *P* < 0.001 η^2^ = 0.5	F = 2.7 *P* = 0.02 η^2^ = 0.1
	RST-ST2 (s)	5.64 ± 0.28	5.50 ± 0.32	5.45 ± 0.18	5.35 ± 0.21			
	RST-ST3 (s)	5.53 ± 0.29	5.49 ± 0.27	5.51 ± 0.27	5.33 ± 0.19			
	RST-ST4 (s)	5.50 ± 0.21	5.55 ± 0.24	5.49 ± 0.22	5.43 ± 0.21			
	RST-ST5 (s)	5.61 ± 0.28	5.53 ± 0.27	5.5 ± 0.27	5.41 ± 0.21			
	RST-ST6 (s)	5.67 ± 0.29	5.63 ± 0.23	5.64 ± 0.29	5.53 ± 0.27			
	RST-ST7 (s)	5.76 ± 0.31	5.61 ± 0.29	5.61 ± 0.27	5.45 ± 0.22			
	RST-ST8 (s)	5.82 ± 0.34	5.59 ± 0.27	5.65 ± 0.32	5.6 ± 0.27			
	RST-ST9 (s)	5.8 ± 0.35	5.68 ± 0.31	5.60 ± 0.28	5.54 ± 0.27			
	RST-ST10 (s)	5.91 ± 0.41	5.8 ± 0.28	5.72 ± 0.29	5.68 ± 0.28			

Abbreviations: ANOVA, analysis of variance; CON, no coffee; LDEC, low-dose espresso coffee; HDEC, high-dose espresso coffee; PLA, decaffeinated espresso coffee; RST, repeated sprint test; RST-ST (1-10), time of the first to tenth sprints during SPT; SD, standard deviation; SPT, soda pop test.

**FIGURE 3:**
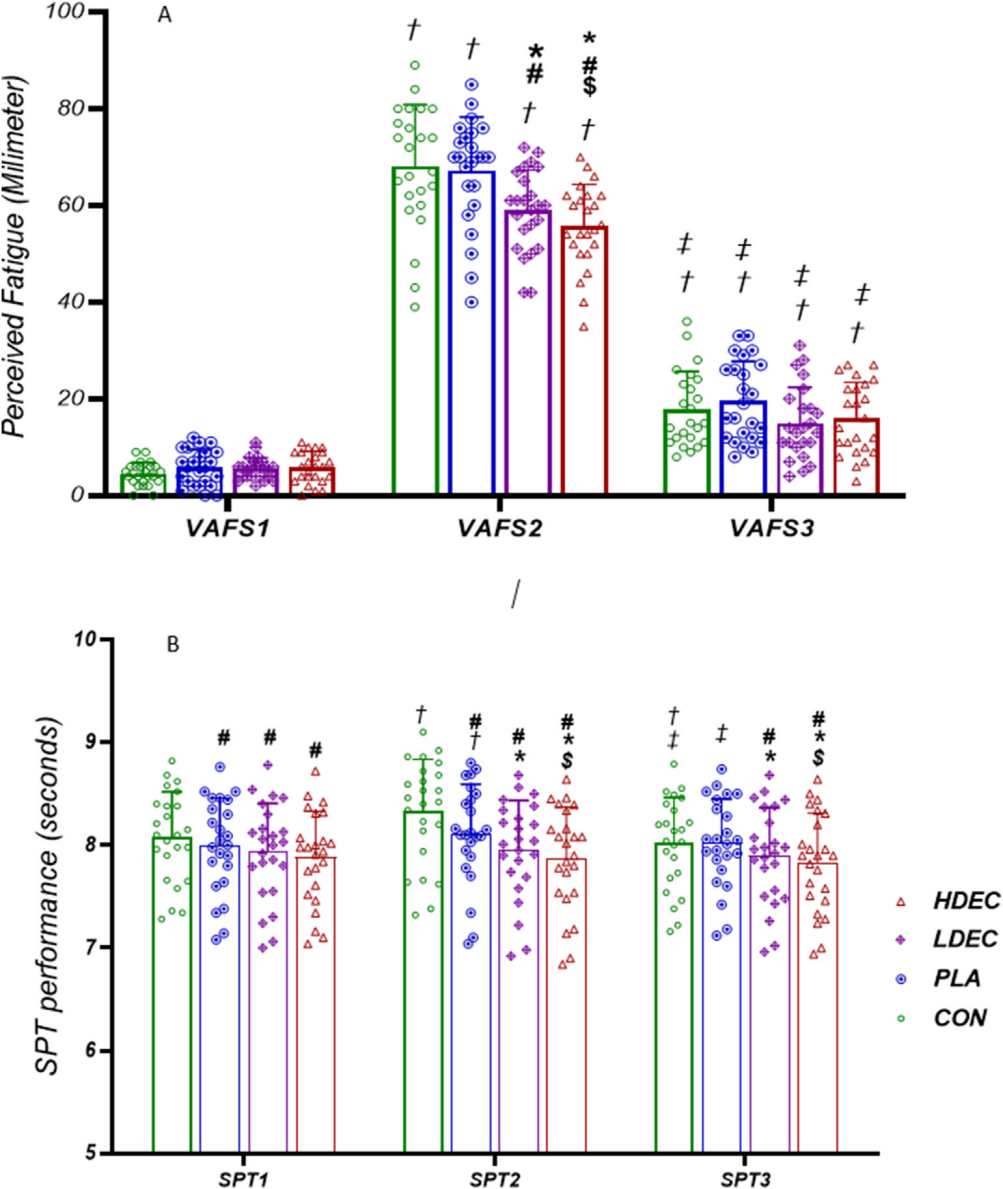
(A) Visual analog fatigue scale (VAFS) and (B) soda pop test performance (SPT) of different conditions at the baseline (VAFS1 and SPT1), immediately after the repeated sprint test (VAFS2 and SPT2) and 5 min after that (VAFS3 and SPT3). ∗Significant difference with PLA. ^#^Significant difference with CON. ^†^Significant difference with baseline value. ^‡^Significant difference with immediately after the repeated sprint test. *P* < 0.05. CON, no coffee; HDEC, high-dose espresso coffee; LDEC, low-dose espresso coffee; PLA, decaffeinated espresso coffee.

Immediately after RST, perceived fatigue increased significantly in all conditions compared with baseline (*P* < 0.001) (Figure 3). In addition, 5 min after RST compared to immediately after RST, perceived fatigue showed a significant decrease in all conditions (*P* < 0.001). However, 5 min after RST compared to baseline, perceived fatigue was still significantly higher in the studied conditions (*P* < 0.001) (Figure 3).

The comparison between the conditions at each time point showed that the baseline perceived fatigue was not significantly different between any of the studied conditions (*P* > 0.05) (Figure 3). Immediately after RST, perceived fatigue of CON was significantly increased compared to HDEC (*P* < 0.001) and LDEC (*P* < 0.001) (Figure 3). However, there was no significant difference in perceived fatigue between PLA and CON immediately after RST (*P* > 0.05). Also, immediately after RST, perceived fatigue was significantly increased in the PLA condition compared to LDEC (*P* < 0.001) and HDEC (*P* < 0.001) (Figure 3). In addition, immediately after RST, the perceived fatigue of LDEC compared to HDEC showed a significant increase (*P* = 0.001). Five minutes after RST, there was no significant difference between any of the conditions in perceived fatigue (*P* > 0.05) (Figure 3).

### SPT performance

Two-way (time × condition) repeated measures ANOVA showed that the main effect of condition (*P* < 0.001), time (*P* < 0.001), and interaction (*P* < 0.001) on SPT performance was significant (Table 1).

The performance of SPT decreased significantly in PLA (*P* = 0.001) and CON (*P* < 0.001) immediately after RST compared to baseline; however, no significant changes were observed in HDEC and LDEC (*P* > 0.05) (Figure 3). Five minutes after RST compared to baseline, SPT performance did not change significantly after PLA, LDEC, and HDEC (*P* > 0.05); however, in the control condition, a significant improvement in SPT performance was observed (*P* < 0.001) (Figure 3). Although 5 min after RST compared to immediately after RST, the performance of SPT for LDEC (*P* = 0.068) and HDEC (*P* = 0.70) did not change significantly, but it improved significantly in PLA (*P* < 0.001) and CON (*P* < 0.001) (Figure 3).

The comparisons between conditions at each time point showed that baseline SPT performance was not significantly different between PLA and LDEC (*P* = 0.11) (Figure 3). However, compared to the CON baseline performance of SPT in LDEC (*P* < 0.001), HDEC (*P* < 0.001) and PLA (*P* = 0.001) improved significantly. Also, baseline SPT performance improved significantly after HDEC compared to LDEC (*P* = 0.008). Immediately after RST, the performance of SPT in HDEC (*P* < 0.001), LDEC (*P* < 0.001), and PLA (*P* < 0.001) was significantly increased compared to CON. In addition, SPT performance immediately after RST for HDEC (*P* < 0.001) and LDEC (*P* < 0.001) conditions significantly improved compared to PLA. Furthermore, SPT performance immediately after RST in HDEC significantly improved compared to LDEC (*P* < 0.001) (Figure 3). Five minutes after RST, there was no significant difference in SPT performance between PLA and CON (*P* > 0.999). However, 5 min after RST, both LDEC (*P* = 0.004) and HDEC (*P* < 0.001) showed a significant increase in SPT performance compared to CON. In addition, 5 min after RST, LDEC (*P* = 0.001) and HDEC (*P* < 0.001) showed a significant increase in SPT performance compared to PLA. Comparison of LDEC and HDEC also showed that 5 min after RST, SPT performance increased significantly in HDEC compared to LDEC (*P* = 0.002) (Figure 3).

### Overall performance of the RST

One-way repeated measures ANOVA showed that the main effect on RST-TT, RST-MT, and RST-BT was significant (*P* < 0.001). However, no significant effect was observed on PD (*P* = 0.6) (Table 2).

**TABLE 2:** One-way repeated measures ANOVA test results to determine the main effect of the interventions on the overall performance of RST

Variables	CON (*n* = 24)	PLA (*n* = 24)	LDEC (*n* = 24)	HDEC (*n* = 24)	Condition effect
	Mean ± SD	Mean ± SD	Mean ± SD	Mean ± SD	
RST-BT (s)	5.44 ± 0.21	5.33 ± 0.25	5.33 ± 0.16	5.23 ± 0.17	F = 16.1 *P* < 0.001 η^2^ = 0.4
RST-TT (s)	56.78 ± 2.79	55.92 ± 2.27	55.70 ± 2.26	54.64 ± 1.93	F = 21.7 *P* < 0.001 η^2^ = 0.5
RST-MT (s)	5.68 ± 0.28	5.59 ± 0.23	5.57 ± 0.23	5.46 ± 0.19	F = 21.7 *P* < 0.001 η^2^ = 0.5
RST-PD (%)	4.48 ± 2.77	4.87 ± 2.15	4.40 ± 2.21	4.37 ± 1.69	F = 0.4 *P* = 0.63 η^2^ = 0.02

Abbreviations: ANOVA, analysis of variance; CON, no coffee; HDEC, high-dose espresso coffee; LDEC, low-dose espresso coffee; PLA, decaffeinated espresso coffee; RST, repeated sprint test; RST-BT, RST best time; RST-MT, RST mean time; RST-PD, RST performance decrement; RST-TT, RST total time; SD, standard deviation.

**FIGURE 4:**
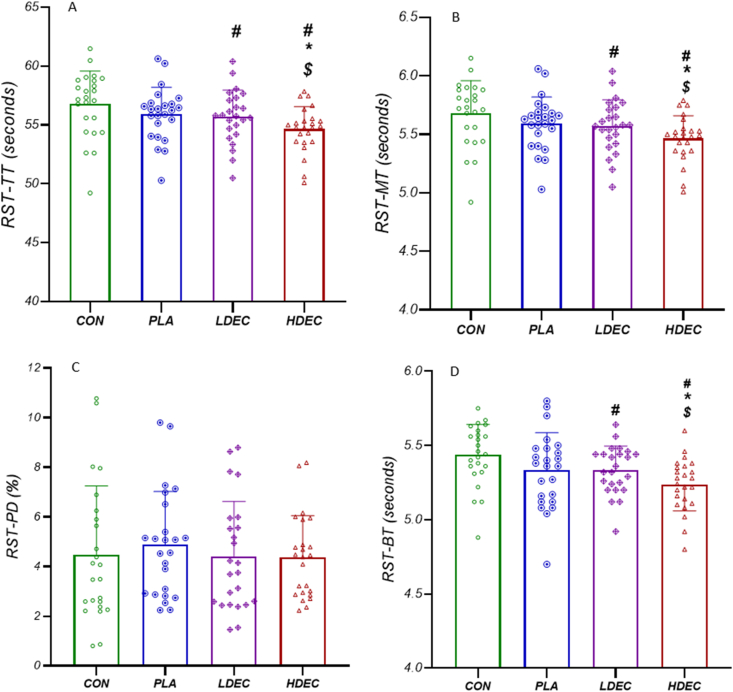
Changes in overall performance of the repeated-sprint test after each condition. (A) The total time of sprints (RST-TT). (B) Mean time of sprints (RST-MT). (C) Performance decrement (PD). (D) Best time between sprints (RST-BT). ∗Significant difference with PLA, ^#^Significant difference with CON, ^$^Significant difference with LDEC. CON, no coffee; HDEC, high-dose espresso coffee, LDEC, low-dose espresso coffee; PLA, decaffeinated espresso coffee.

RST-TT in HDEC significantly improved compared to CON (*P* < 0.001), PLA (*P* < 0.001), and LDEC (*P* < 0.001). In addition, LDEC significantly improved RST-TT compared to CON (*P* < 0.001). Nevertheless, no significant difference was observed in RST-TT between PLA and CON (*P* > 0.05) and between PLA and LDEC (P > 0.05) (Figure 4).

RST-MT in HDEC significantly improved compared to the CON (*P* < 0.001), PLA (*P* < 0.001), and LDEC (*P* < 0.001). In addition, LDEC significantly improved RST-MT compared to CON (*P* < 0.001). However, no significant difference was observed in RST-MT between PLA and CON (*P* > 0.05) and between LDEC and PLA (*P* > 0.05) (Figure 4).

HDEC significantly improved BT-RST compared to CON (*P* < 0.001), LDEC (*P* < 0.001), and PLA (*P* = 0.004). In addition, LDEC significantly improved RST-BT compared to CON (*P* = 0.001). However, no significant difference was observed between LDEC and PLA (*P* > 0.05), and between PLA and CON (*P* = 0.06) in RST-BT (Figure 4).

### Each sprint performance (RST_1_-RST_10_)

Two-way (time × condition) repeated measures ANOVA showed that the main effect of time (*P* < 0.001), condition (*P* < 0.001), and interaction (*P* = 0.02) on RST-ST_1-10_ was significant (Table 1).

**FIGURE 5:**
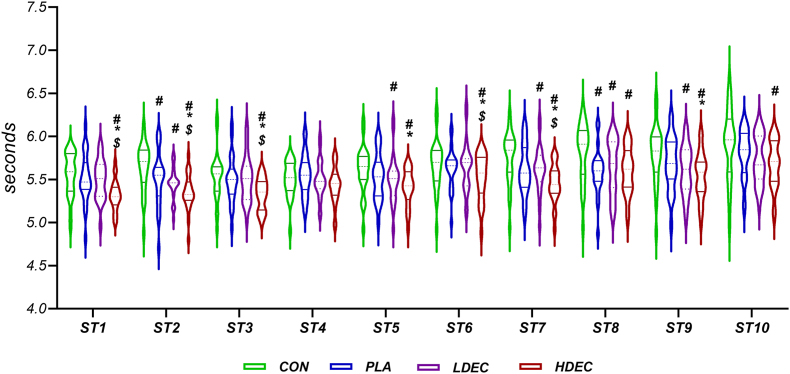
The time of each sprint (ST1-ST10) during the repeated sprint tests. ^$^Significant difference with LDEC, ^#^Significant difference with CON, ∗Significant difference with PLA (*P* < 0.05). CON, no coffee; HDEC, high-dose espresso coffee; LDEC, low-dose espresso coffee; PLA, decaffeinated espresso coffee.

RST-ST_1_ was significantly improved in HDEC compared to CON (*P* < 0.001), PLA (*P* = 0.005), and LDEC (*P* = 0.001). However, no significant difference in RST-ST_1_ performance was observed between other pairs (*P* > 0.05) (Figure 5).

PLA (*P* = 0.018), LDEC (*P* < 0.001), and HDEC (*P* < 0.001) significantly improved RST-ST_2_ compared to CON. No significant difference was observed between LDEC and PLA (*P* > 0.05). However, HDEC showed a significant improvement in RST-ST_2_ compared to PLA (*P* = 0.007) and LDEC (*P* = 0.018) (Figure 5).

RST-ST_3_ showed a significant improvement in HDEC compared to LDEC (*P* < 0.001), CON (*P* = 0.001), and PLA (*P* = 0.001). However, no significant difference in RST-ST_3_ performance was observed between other pairs (*P* > 0.05) (Figure 5).

RST-ST_4_ did not show a significant difference after any of the conditions (*P* > 0.05).

LDEC (*P* < 0.001) and HDEC (*P* < 0.001) significantly improved RST-ST_5_ compared to CON. In addition, RST-ST_5_ was significantly improved in HDEC compared to PLA (*P* = 0.04). However, no significant difference in RST-ST_5_ performance was observed between other pairs (*P* > 0.05) (Figure 5).

RST-ST_6_ showed a significant improvement in HDEC compared to LDEC (*P* = 0.04), CON (*P* = 0.001), and PLA (*P* = 0.03). However, no significant difference in RST-ST_6_ performance was observed between other pairs (*P* > 0.05) (Figure 5).

RST-ST_7_ showed a significant improvement in HDEC compared to LDEC (*P* = 0.02), CON (*P* < 0.001), and PLA (*P* = 0.001). LDEC showed a significant improvement in RST-ST_7_ compared to CON (*P* < 0.001). However, no significant difference was observed between LDEC and PLA (*P* > 0.05), and between PLA and CON (*P* = 0.08) (Figure 5).

RST-ST_8_ showed a significant increase in CON compared to PLA (*P* = 0.001), HDEC (*P* < 0.001), and LDEC (*P* = 0.001). However, no significant difference in RST-ST_8_ performance was observed between other pairs (*P* > 0.05) (Figure 5).

RST-ST_9_ showed a significant improvement in HDEC compared to CON (*P* < 0.001) and PLA (*P* = 0.01), but there was no significant difference compared to LDEC (*P* = 0.07). LDEC also significantly improved RST-ST_9_ compared to CON (*P* = 0.001). However, no significant difference was observed between LDEC and PLA (*P* > 0.05). In addition, no significant difference was observed in RST-ST_9_ between PLA and CON (*P* > 0.05) (Figure 5).

RST-ST_10_ in HDEC improved significantly compared to CON (*P* = 0.008). However, no significant difference in RST-ST_10_ performance was observed between other pairs (*P* > 0.05) (Figure 5).

## Discussion

The findings of this study showed that oral ingestion of espresso coffee (high and low doses) compared to CON or PLA conditions could improve performance in RSTs and eye–hand coordination. However, the higher dose of espresso coffee (double shot) showed a more profound effect compared to the low dose (single shot).

Perceived fatigue increased significantly in all trials immediately after RST compared to baseline. However, 5 min after RST, perceived fatigue reduced compared to immediately after RST but was still higher than baseline. The perceived fatigue level immediately after RST indicated a significant difference between the conditions such that it was lower after coffee consumption than PLA and CON. Some previous studies have also confirmed the positive effect of caffeine/coffee on the improvement of perceived fatigue immediately after high-intensity exercises [29,41,42], whereas others have not shown a significant effect [27,28,43,44]. Differences in instruments and timing of perceived fatigue measurement, exercise protocols, and coffee/caffeine supplementation strategies may contribute to the heterogeneity of the results. It should also be noted that although studies have not shown a significant effect of caffeine/coffee on reducing perceived fatigue or perceived effort after exercise protocols [27,28,43], they have shown an improvement in exercise performance [27,28,43,44], which itself indicates the effect of delaying fatigue by caffeine.

Increased perceived fatigue after RST can be due to central and peripheral fatigue factors. For example, muscle glycogen has been reported to decrease after RST [45], which could contribute to fatigue during high-intensity sprint exercises [45,46]. In addition, during high-intensity exercise, metabolites such as adenosine, H^+^, bradykinin, and extracellular K^+^ increase [45,[47], [48], [49]], which can cause fatigue by inhibiting the central nervous system [48,50] and disrupting the contractile function of skeletal muscles. Also, it has been suggested that central motor drive is dependent on the rate of development of peripheral fatigue and increased afferent feedback (III/IV afferents) inhibits the brain’s ability to recruit motor units [47,50]. One of the most important mechanisms to explain the reduction of fatigue caused by coffee consumption is the antagonistic effect of caffeine on adenosine receptors. Adenosine levels increase during muscle contractions and cause fatigue and pain by binding to adenosine receptors [27,51]. However, coffee ingestion can reduce the negative effects of adenosine on exercise performance by increasing the plasma levels of caffeine through the antagonist effect on the A1/A2 adenosine receptors [52,53]. Also, the antagonistic effect of caffeine on the adenosine receptor may prevent the reduction of the firing rate of motor units, which improves muscle endurance during high-intensity activities [14,51]. This anti-fatigue effect of caffeine can be one of the possible factors related to improving the overall (RST-TT, RST-MT) and individual (RST-ST_1_ to RST-ST_10_) performance in RST, especially after the HDEC condition. Interestingly, perceived fatigue at 5 min after RST showed a significant decrease compared to immediately after RST without any difference between the different conditions, which may be due to the rapid clearance of adenosine (half-life 0.6–10 s) [54]. However, because adenosine levels were not measured in the present study, its measurement is suggested for more clarification.

The improvement of RST performance after coffee consumption in the present study is supported by some previous studies [28,43,44]. For example, Karayigit et al. [28] showed that low- to moderate-dose caffeine supplementation improved RST performance (12 × 4 s all-out sprints interspersed with 20 s active recovery on a cycle ergometer) in female team sports players [28]. Another study on male team sports players showed that coffee consumption can improve the performance of the first 3 sprints in the intermittent sprint cycling test (12 × 4 s all-out sprints separated by 90 s active recovery at 60 W on a cycle ergometer) [43]. Improvements in repeated sprint performance after caffeine consumption have also been confirmed in male rugby players [55] and competitive male cyclists [56]. However, a meta-analysis in 2013 also reported that caffeine ingestion has no significant effect on the repeated sprint performance of team athletes compared to placebo. It should be noted that in this meta-analysis, the role of caffeine dose, timing, and the method of supplementation was not considered. In addition, Clark et al. [57] reported that coffee consumption (providing 3 mg caffeine/kg body mass) had little effect on repeated sprint performance. However, the authors suggested that trained athletes may be more suitable for the ergogenic effects of caffeine/coffee in high-intensity exercises [57]. Consistent with this hypothesis, another study suggested that a moderate dose of caffeine (3–6 mg/kg) in trained athletes is likely to increase RST ability in different sports populations [27]. Therefore, the improvement of RST performance in HDEC compared to other conditions (LDEC, PLA, and CON) may be due to higher levels of caffeine as well as the training status of the participants. According to the caffeine levels in each shot of espresso, it seems that under HDEC (double shot, 160 mg caffeine) conditions, sufficient amounts of caffeine are provided to affect RST performance compared to LDEC (single shot, 80 mg caffeine). In this regard, a study has also suggested that the levels of caffeine used are a mediator in the effectiveness of beverages on RST performance [58]. Considering that a reduction in perceived fatigue compared to CON and PLA has been found immediately after RST, HDEC, and LDEC, the improvement of RST performance can be related to the mechanisms of caffeine’s effect on fatigue. Furthermore, mechanisms that directly affect muscle contractile function [59,60] may play a role in improving RST performance for HDEC and LDEC. In this regard, it has been reported that caffeine can improve muscle fiber contractility by facilitating sarcoplasmic calcium release and activity of the sodium-potassium ATPase enzyme [[61], [62], [63], [64], [65]]. It has also been shown that caffeine can reduce plasma and muscle interstitium K^+^, have a positive effect on neuromuscular transmission [65], and decrease phosphocreatine and H^+^ accumulation [66], which can justify the present study findings regarding the improvement of RST (last repetitions). There is also evidence that suppressing inhibitory mechanisms (activation of small-diameter group III/IV afferents) caused by caffeine can be associated with improved performance in subsequent exercise [64]. Therefore, the improvement of RST-BT, RST-MT, and RST-TT performance as well as individual sprints in the last part of RST after coffee consumption can be explained according to these mechanisms. However, more comprehensive and controlled studies are needed.

Consistent with the findings of the present study, it has been shown that caffeine can have positive effects on eye–hand coordination [[67], [68], [69]]. In addition, some other studies have also shown the positive effects of caffeine on neuromuscular coordination in animals [[70], [71], [72], [73]] and humans [69]. However, several studies have also shown that acute caffeine consumption leads to decreased performance in sensitive and hand steadiness-dependent tasks [[74], [75], [76], [77], [78]]. It should be noted that these inconsistent studies have often used tests based on fine motor patterns (requiring fine finger movements and high levels of accuracy), and none of them have used SPT to determine eye–hand coordination. Therefore, the inconsistency of the results could be due to differences in the method of measuring eye–hand coordination, the level of complexity of the tasks, or their interaction with the caffeine dose. There is some debate about the effect of caffeine on simple compared with complex tasks [51]. Einöther and Giesbrecht [79] showed that caffeine has a positive effect on both, such that simple tasks can be affected with lower doses (0.2–5.5 mg/kg) but more complex tasks require higher doses (0.75–5.5 mg/kg). Also, the strategy of caffeine consumption or other methodological factors may affect the heterogeneity of the results. For example, Toktaş et al. [78] reported that acute coffee mouth-rinsing (compared with oral ingestion of coffee in the present study) has no significant effect on eye–hand coordination. In this study, recreationally active female subjects were administered coffee mouthwash only once, which did not increase blood plasma levels of caffeine sufficiently to improve cognitive and physical performance. Also, it should be noted that baseline SPT performance in PLA improved compared to CON, and there was no significant difference between LDEC and HDEC. Therefore, the improvement of baseline SPT performance in LDEC or HDEC can also be due to the placebo effect. Some previous studies have also confirmed that the ergogenic or physiologic effect of the placebo can be comparable to caffeine [[80], [81], [82], [83]], but others have not reported a significant effect [84]. In this study, decaffeinated coffee was used as a placebo, which may partially explain the placebo effect on performance improvement. So, participants’ knowledge of the ergogenic properties of caffeine may have influenced the results observed in the placebo condition. It has also been reported that a placebo enhanced performance when participants assumed that the substance they were consuming was caffeine [82,83]. Some research has also shown that participants who consciously consumed caffeine increased their performance, and it seemed that the belief that they were consuming caffeine was necessary to obtain an ergogenic effect [85,86].

Another finding of the present study was the negative effect of muscle fatigue caused by RST (immediately after RST) on eye–hand coordination in CON and PLA conditions, which was inhibited by coffee consumption in a dose-dependent manner. According to these findings, previous studies have also indicated that lower-body extremity fatigue can reduce upper-body performance in motor tasks with high neuromuscular demands [87]. In addition, it has previously been confirmed that the muscles of the lower body play a significant role in improving the functional characteristics of the upper limbs (force production, reaction time, and velocity) [[88], [89], [90], [91]]. Therefore, the decrease in SPT performance in CON and PLA may be due to the negative effects of lower body fatigue (caused by RST). No study was found that investigated the interaction of caffeine supplementation with the negative effects of lower-body fatigue on upper-body performance. However, the improvement in SPT performance immediately after RST following coffee consumption can be explained by some previous evidence. For example, caffeine/coffee has been reported to improve eye–hand coordination performance in sleep deprivation-induced fatigue state [68]. Another study reported that caffeine reduced ethanol-induced incoordination in mice [71]. It was suggested that the positive effect of caffeine on motor coordination is due to the antagonistic effect of caffeine with adenosine receptors [51,71,78]. Caffeine improves visual information processing [51,92,93], which may also contribute to increased SPT performance. In addition, it should be noted that in addition to attention, accuracy, and concentration, SPT performance is also affected by the velocity of muscle contraction and hand movement or hand agility. Therefore, the mechanisms that affect the activation and rate of neuromuscular conduction as well as the cross-bridge cycle in the muscle contraction cycle may play a role in improving SPT performance after RST. These findings may be important for basketball players, as coffee/caffeine reduces the negative effect of fatigue due to repetitive sprints on eye–hand coordination, which in turn improves overall basketball performance. In this regard, previous studies have also confirmed the positive effect of acute caffeine/coffee consumption on improving basketball performance [94,95]. However, some studies have also shown that caffeine consumption does not have a significant effect on improving basketball-specific performance [96,97]. However, these studies have not investigated the role of caffeine on specific basketball performance in the fatigue state. Therefore, according to this conflicting evidence, evaluating the effect of caffeine/coffee on basketball-specific skills needs more clarification. The strength and novelty of this study was the measurement of coffee effect during fatigue status. Limitations of this study included a small number of participants and not measuring blood caffeine concentration. In addition, due to the possible effect of the menstrual cycle on measurements, male participants were included in the present study; however, the response of females to similar interventions may be considered in future studies.

Taken together, this study showed that fatigue caused by RST negatively affects eye–hand coordination performance. However, consuming coffee can reduce the negative effects of fatigue on eye–hand coordination. Furthermore, coffee improved RST performance and reduced perceived fatigue dose-dependently in male basketball players.

## Author contributions

The authors’ responsibilities were as follows – MKJ, AN: conceptualization; MKJ, MHA, AN: methodology; AN: software; AN: formal analysis; AHA, AN: investigation; MKJ, MHA, MH: resources; AN, AHA: data curation; AN: wrote the original draft; MKJ, AN: reviewed and edited the manuscript; MKJ: supervision; AN: project administration; AN, MHA: funding acquisition; and all authors: read and approved the final manuscript.

## Conflict of interest

The authors report no conflicts of interest.

## Funding

The authors reported no funding received for this study.

## Data availability

Data described in the manuscript will be made available upon request.
